# Habitat usage of Daubenton's bat (*Myotis daubentonii*), common pipistrelle (*Pipistrellus pipistrellus*), and soprano pipistrelle (*Pipistrellus pygmaeus*) in a North Wales upland river catchment

**DOI:** 10.1002/ece3.5085

**Published:** 2019-03-25

**Authors:** Victoria L. G. Todd, Laura D. Williamson

**Affiliations:** ^1^ Ocean Science Consulting Ltd. Dunbar UK; ^2^ School of Media Arts and Technology Southampton Solent University Southampton UK

**Keywords:** elevation, habitat use, *Myotis daubentonii*, *Pipistrellus pipistrellus*, *Pipistrellus pygmaeus*, riparian, Wales

## Abstract

Distributions of Daubenton's bat (*Myotis daubentonii*), common pipistrelle, (*Pipistrellus pipistrellus*), and soprano pipistrelle (*Pipistrellus pygmaeus*) were investigated along and altitudinal gradient of the Lledr River, Conwy, North Wales, and presence assessed in relation to the water surface condition, presence/absence of bank‐side trees, and elevation. Ultrasound recordings of bats made on timed transects in summer 1999 were used to quantify habitat usage. All species significantly preferred smooth water sections of the river with trees on either one or both banks; *P. pygmaeus* also preferred smooth water with no trees. Bats avoided rough and cluttered water areas, as rapids may generate high‐frequency echolocation‐interfering noise and cluttered areas present obstacles to flight. In lower river regions, detections of bats reflected the proportion of suitable habitat available. At higher elevations, sufficient habitat was available; however, bats were likely restricted due to other factors such as a less predictable food source. This study emphasizes the importance of riparian habitat, bank‐side trees, and smooth water as foraging habitat for bats in marginal upland areas until a certain elevation, beyond which bats in these areas likely cease to forage. These small‐scale altitudinal differences in habitat selection should be factored in when designing future bat distribution studies and taken into consideration by conservation planners when reviewing habitat requirements of these species in Welsh river valleys, and elsewhere within the United Kingdom.

## 1 INTRODUCTION

Bats are unusual animals with arcane ecology that engenders human emotions ranging from apathy to fear. Human populations tend to aggregate around the bodies of water, where insect abundance is high (Cole, Brocklehurst, Robertson, Harrison, & McCracken, 2015; Dreyer et al., 2015), and since these regions also serve as important foraging habitats for many species of insectivorous bat (e.g., Grindal, Morissette, & Brigham, 1999; Williams, O'Farrell, & Riddle, 2006), human–bat interactions are unavoidable. Consequently, bats that feed on aquatic biota in riparian regions are especially vulnerable to anthropogenic impacts, in addition to local environmental factors such as changes to physical habitat and water chemistry, as well as larger‐scale pressures such as condition of riparian corridor and land use of the catchment (e.g., Bedoya, Manolakos, & Novotny, 2011; Salvarina, 2016). Bats are known to be effective bioindicators of ecosystem health (Jones, Jacobs, Kunz, Willig, & Racey, 2009; Park, 2015; Russo & Jones, 2015; Scott, McLaren, Jones, & Harris, 2010); therefore, a good understanding of bat habitat requirements is fundamental to the implementation of successful riparian management regimes (e.g., Ancillotto et al., 2017; Millon, Colin, Brescia, & Kerbiriou, 2018).

Foraging activity in bats is not uniform, with individuals favoring certain environmental characteristics over others. For example, Warren, Waters, Altringham, and Bullock (2000) and Todd and Waters (2017) found that foraging of both Daubenton's bats (*Myotis daubentonii*) and common pipistrelle (*Pipistrellus pipistrellus*) in upper Wharfedale (Yorkshire Dales National Park, UK) preferred river sections with smooth water and trees on both sides. This is in agreement with Lundy and Montgomery (2010), who also found that *M. daubentonii *favor smooth water areas.

Elevation is also thought to impact bat abundance and foraging efficiency. Grindal et al. (1999) found that foraging activity of bats in a riparian area of British Columbia decreased with increasing elevation, as did insect availability. Todd and Waters (2007) found that, at the same location as this study (Lledr River, Conwy, North Wales, UK), *M. daubentonii *switched feeding strategy with elevation. At the lower elevation site, bats’ primary hunting method was aerial hawking, regardless of aerial prey density, but at higher elevations, a switch to gaffing was observed. Dunn and Waters (2012) also found that the activity of *P. pipistrellus* and *Pipistrellus pygmaeus* declined with increasing elevation. Elevation has been found to play a significant role in bat community structure and could potentially be used to predict assemblage structure due to relationships between plants and elevation (Capaverde et al., 2018). Elevation may also influence sexual segregation—with male *M. daubentonii* recorded at higher elevations (Nardone et al., 2015); consequently, even small‐scale changes in the environment can influence bat foraging ability (Salvarina, 2016).

Habitat modifications have been correlated with bat population declines on a number of occasions (e.g., Millon et al., 2018; Russo & Ancillotto, 2015), and identification of factors that influence bats substantially requires collection of quantitative data on the significance of each habitat type. Difficulties of studying flying bats at night in nonlinear habitats mean that published studies are limited in both size and number; consequently, there are numerous knowledge gaps for many vulnerable species. Fortunately, *M. daubentinii*, *P. pipistrellus*, and *P. pygmaeus* are known to forage along linear landscape elements, such as rivers and tree lines (Downs & Racey, 2006; Verboom & Huitema, 1997), rendering them relatively easy species to study through the night.

Data presented here were collected 20 years ago and so provide a useful baseline for which future studies can be compared to investigate potential effects of a changing climate. Alterations to bat detection, habitat use, reproduction, and migrations have been reported with increasing effects of climate change (Adams, 2010,2018; Rebelo, Tarroso, & Jones, 2010; Sherwin, Montgomery, & Lundy, 2013); therefore, understanding environmental drivers linked to detections of species is vital for the informed development of management strategies. Results of this study can have significant management implications for these species in this region, and elsewhere in upland riparian valleys.

This paper investigates the effects of three main factors thought to affect bat distributions: (a) water characteristics, (b) bank‐side trees, and (c) elevation. We examine detections of *M. daubentonii*, *P. pipistrellus*, and *P. pygmaeus* in relation to these factors along a river gradient, as well as their time of emergence. Based on previous studies of habitat usage (Todd & Waters, 2017) and detections along altitudinal gradients (Walsh & Harris, 1996), we predict that bats are more likely to be detected along smooth water sections of river with trees along the banks and also that detections will decrease with increasing elevation.

## 2 MATERIALS AND METHODS

### 2.1 Study area

**Figure 1:**
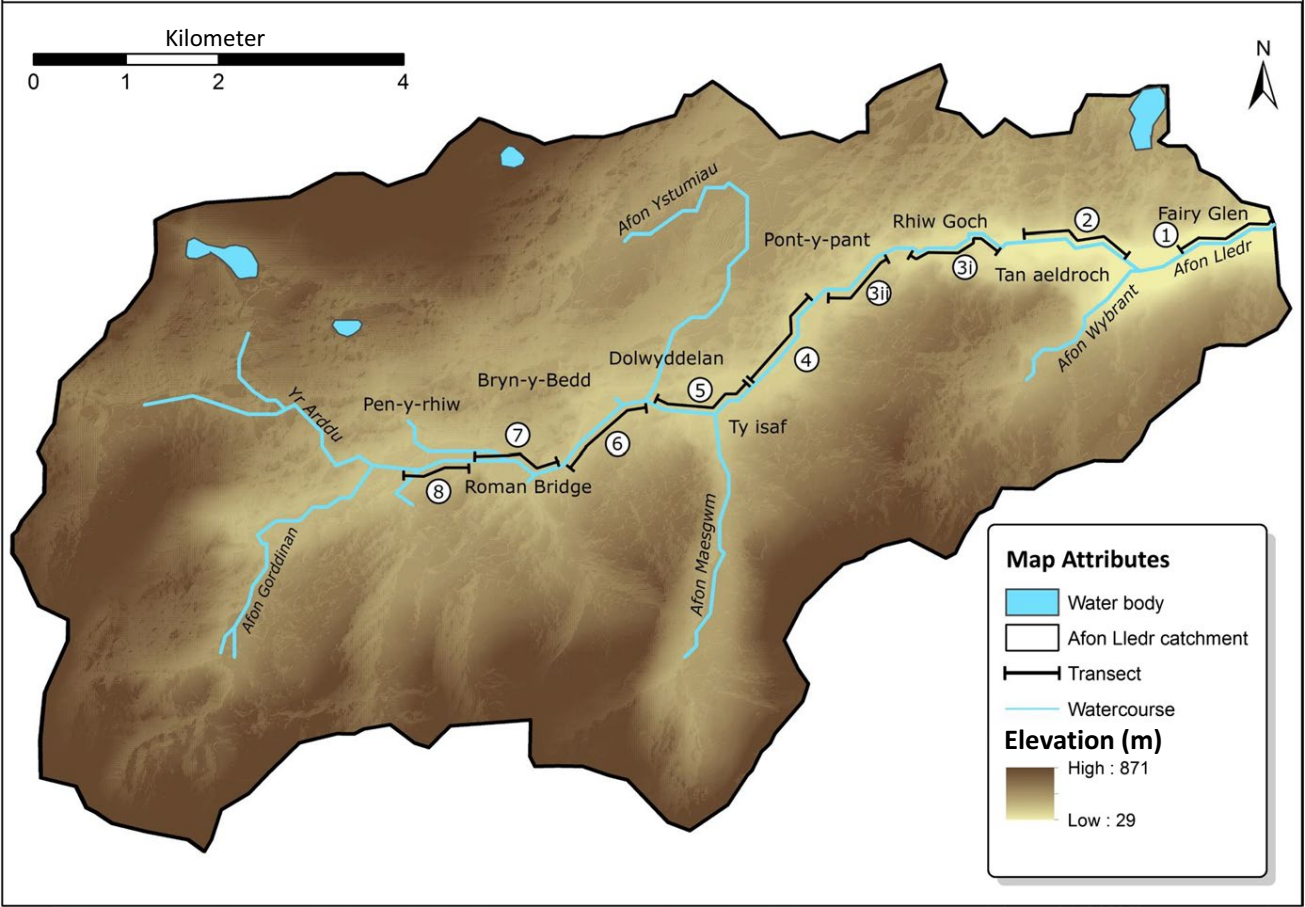
Map of transects in Lledr valley

Data were collected between 14 July and 3 August 1999 along an 8.65 km stretch of the river (Afon) Lledr which runs through the Lledr Valley, North Wales, UK. The study area was located between Fairy Glen (53º03′N 3º48′W) and Pen‐y‐rhiw (53º02′N 3º56′W; Figure 1).

The upper reaches of the Lledr are characterized generally by smooth water with few trees. Beyond Roman Bridge, the river is bordered mostly by trees (mainly alder, *Alnus glutinosa*), thick riparian vegetation, and littered with large boulders and rapids. At Dolwyddelan, the river smooths out again and trees are scarce (similar to upper regions), but beyond the bridge at Pont‐y‐Pant it narrows, flowing through several gorges bordered by trees and riparian shrubs all the way to the Conwy confluence beyond Fairy Glen. About 50% of the Lledr is bordered by riparian woodland, especially in the mid‐ and lower reaches. The Lledr is generally of similar width (*ca*. 6–10 m) along its entire length, with a few narrow stretches between 3 and 4 m. Water depth in summer rarely exceeds two meters, typically being 0.3–0.8 m deep.

### 2.2 Habitat mapping

Length of the Lledr was mapped for the nine different habitat types presented in Table 1, as in the studies of Warren et al. (2000) and Todd and Waters (2017). Where possible, length of each habitat was paced and converted to meters; however, for some areas, pacing was not possible due to the difficulty of climbing over boulders and fences; therefore, for these areas, individual habitats were measured to the nearest millimeter from the 1:25,000 map enlarged to 1:12,000.

**Table 1:** Major physical features of the Afon Lledr according to Warren et al. (2000) and Todd and Waters (2017)

Major category	Subcategory	Habitat no.
Smooth water surface	Trees present on both banks	1
	Trees present on one bank	2
	No trees on either bank	3
Rapid water surface	Trees present on both banks	4
	Trees present on one bank	5
	No trees on either bank	6
Cluttered water surface	Trees present on both banks	7
	Trees present on one bank	8
	No trees on either bank	9

Note

Trees were more than 5 m high with branches touching each other, that is, no gaps between adjacent trees; each tree was more than 5 m in width including branches.

Water was classified as smooth, rapid, or cluttered. Smooth water was defined as lacking white water or riffles, rapid water had white water and heavy riffles, and cluttered water contained projecting rocks and riffles.

### 2.3 Bat detections

Due to the precarious nature of the river borders, scattered usually with large boulders, barbed wire fences, and impenetrable scrub barriers, it was impossible to select transects of a set length. Consequently, eight transects of varying length were selected which could each be walked in 30 min (Table 2).

**Table 2:** Location of transects along the Afon Lledr, North Wales

T no.	Location and direction of transect	Alt. (m)	ΔAlt (m)	Dist. (m)	TL (m)	Time (min)
1.	Fairy Glen (NE‐SW)	40	—	—	1,000	30
2.	Tan aeldroch (W‐E)	104	64	900	1,063	30
3i	Rhiw Goch (W‐E)	130	26	750	613	15
3ii	Pont‐y‐pant (NE‐SW)	130	0	430	725	15
4.	Dolwyddelan (W‐NE)	164	34	0	1,313	30
5	Ty isaf (NE‐W)	164	0	0	1,063	30
6	Bryn‐y‐Bedd (E‐SW)	180	16	610	875	30
7	Roman Bridge (W‐E)	207	27	400	975	30
8	Pen‐y‐rhiw (E‐W)	210	3	0	1,025	30

Notes

Transects in the order of elevation along the river from downstream to upstream. All elevations are expressed in meters above mean sea level.

ΔAlt. (m): elevation difference between transects; Alt. (m): elevation of transect; Dist. (m): distance between transects; T no.: transect number; time (min): duration of transect; TL (m): transect length.

Transect three (Rhiw Goch and Pont‐y‐pant) was divided into two 15‐min transects (3i and 3ii) because part of the area was bisected by a forest which forced the observer too far away from the river to detect reliable signals with the bat detector. For treatment of the data and comparison of numbers of bat passes on the outgoing and return transects, transect 3i was treated as the outgoing transect (30 min total comprised of 15 min outgoing and 15 min return) and transect 3ii (30 min total) as the return.

Each transect was walked by the same trained observer twice commencing 14 July and terminating on 3 August 1999 totaling 16 nights for eight transects. Observations approximately covered bat lactation period of species concerned (Swift, 1980). At the transect location, the bat detector was switched on from sunset onwards (21:30 BST), to identify the first bat species to arrive at the transect location. Transects were selected randomly and were walked at a constant pace, commencing 30 min after sunset, on evenings with wind ≤2 ms^−1^, with temperatures ranging from 10–21°C without heavy rain, when bat activity may be suppressed (Turner, 2002).

The bat detector (Tranquillity II, Cheltenham) was always held facing the river to ensure all bat passes were recorded. Detection of *Myotis* and *Pipistrellus *bats using this detector are usually limited to 20 m or less (Adams, Jantzen, Hamilton, & Fenton, 2012), which ensures bats are only detected in the transect areas and not adjacent habitats. The highly directional response of similar designs of detector (Waters & Walsh, 1994) ensured that bats foraging over the river would not be detected at neighboring locations, and only bats using the river would be recorded. The detector, configured to record 40 ms in time expansion mode, was connected via the right channel to a stereo cassette recorder (Genexxa^®^ SCR‐59). The high‐frequency output from the detector was time expanded (10×) for computer analysis as recommended by Jones, Vaughan, and Parsons (2000). The bat detector microphone had a frequency response of ±20 dB, 12–200 kHz and the cassette recorder roughly ±3 dB, 400 Hz–12 kHz (Genexxa were unable to provide exact specifications). Recordings were made on 90‐min normal position tapes (TDK^®^, Luxembourg D‐IEC/type I). The tape recorder was held in the other hand together with the map of the transect for the identification of habitats. Each new habitat passed (together with time) was dictated into the internal microphone on the left channel of the tape recorder using identifiable distance markers such as houses, walls, and bridges. When the transect was completed in one direction, it was walked in reverse commencing 1 hr after sunset. This method investigated the influence of start time on bat distributions. The transect was then replicated the following night.

Spectrographic analysis of time‐expanded calls was performed initially using a shareware dual‐channel audio spectrum analyzer (Spectrogram version 4.2.12 for Windows 95) on a PC. Spectrograms of calls were constructed using a 512‐point Fast Fourier Transform (FFT) on a scope display in real time. Bat species identification was confirmed by analyzing associated time‐expanded audio sequence using Batsound (Petersson Electronic) on a PC and observing the call spectrogram (512‐point FFT, Hamming Window). Calls of *M. daubentonii* were separated from those of *Pipistrellus* species by the lower terminal frequency and the lack of a constant frequency tail at the start of approach phase. Calls between the two phonic forms of pipistrelle (*P. pipistrellus*, *P. pygmaeus*) and potential, but unlikely Nathusius’ pipistrelle (*Pipistrellus nathusii*) were distinguished easily from their echolocation calls, as per (Jones & van Parijs, 1993; Rachwald, Bradford, Borowski, & Racey, 2016; Vaughan, Jones, & Harris, 1997). While separation of echolocation calls of *M. daubentonii* from those of other *Myotis* species is problematic (Walters et al., 2012), previous harp netting at this site has shown that *M. daubentonii* was by far the most common *Myotis* species (V. L. G. Todd, unpublished results), therefore all *Myotis* calls are assumed to be from *M. daubentonii*. Due to the excellent signal to noise ratio in recordings during this study, no filters were required to be used to distinguish echolocation calls.

For analysis of the transect data, bat passes for both outgoing and return transects were used. For investigation into numbers of bats present at each elevation, only data for return transects were used, as bats on outgoing transects were likely to be commuting rather than foraging.

### 2.4 Data analysis

Outgoing and return transects were not pooled for analysis, as there may have been a danger of recording the same bat on the return transect as on the outgoing, if for example, some bats were territorial or had fixed feeding sites; consequently, outgoing and return transects were treated separately. Since length and representation of habitats varied within and between transects, bat passes were corrected for length (i.e., numbers of bat passes per habitat length). Means and standard deviations (*SD*) of pooled numbers of *M. daubentonii*, *P. pipistrellus*, and *P. pygmaeus* passes per km of each habitat type were calculated (Figure 4).

Comparisons of numbers of bats on outgoing and return transects were tested using paired *t *tests. Non‐normally distributed data (which included most bat pass counts) were normalized by (*n* + 1) log_10_ transformations. Data were expressed as total number of bats recorded in each habitat type corrected for habitat length (i.e., number of bats per km). Length rather than area was considered appropriate, as the river did not vary to any large extent in width along its length. A chi‐square contingency table was constructed to test for association of *M. daubentonii*, *P. pipistrellus,* and *P. pygmaeus* recorded in the various habitat combinations with what would be expected for a random distribution. Bonferroni confidence intervals were constructed at the *p* < 0.05 level (Neu, Byers, & Peek, 1974) to determine which habitats were used significantly more or less in proportion to availability as in the studies of Walsh, Harris, and Hutson (1995), Warren et al. (2000), and Todd and Waters (2017). Bonferroni confidence intervals were calculated on a custom written DOS program.

## 3 RESULTS

### 3.1 Habitat mapping

With possible exceptions of cluttered water with trees on one side or no trees (habitats eight and nine respectively), all habitats along the Lledr were well represented (Table 3). Smooth water without trees (habitat three) closely followed by smooth water with trees on one side (habitat two) and then smooth water with trees on both sides (habitat one) were the most well‐represented habitat types in the study area with rapids and cluttered areas covering fewer river stretches.

**Table 3:** Total lengths (km) of each habitat category within the 8.65 km study area. Numbers in brackets refer to habitat type

	Water characteristics						Total
	Smooth		Rapid		Cluttered		
Trees							
Present both sides	1.38	(1)	0.99	(4)	0.94	(7)	3.31
Present one side	2.11	(2)	0.24	(5)	0.14	(8)	2.49
Not present	2.34	(3)	0.40	(6)	0.13	(9)	2.87
Total	5.83		1.63		1.21		

**Figure 2:**
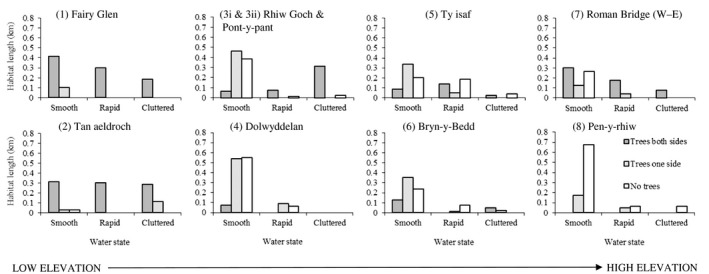
Transects (1)–(8) total length of all nine habitat types present (per transect) in the entire study area from low (Fairy Glen) to high (Pen‐y‐rhiw) river sections

On examination of the proportion of habitats available to bats at different elevations (Figure 2), no single transect contained the full range of habitat types, the most representative being transect five (Ty isaf), and the least representative transect one (Fairy Glen). There were more trees present lower down the river valley than higher up, and more varieties of water state with more rapids and cluttered water (Figure 2). In the midriver section, trees were present mainly on one side, with many smooth water and few cluttered and rapid habitats. At transect seven, there was still a fair proportion of smooth water with trees on either one or neither bank, but at the highest point along the river (transect 8), there was a high proportion of smooth water, but an almost complete absence of trees and cluttered and rapid water habitats.

### 3.2 Bat detections

Three main species of bat were recorded at all elevations: *M. daubentonii*, *P. pipistrellus,* and *P. pygmaeus*. *Pipistrellus pygmaeus *was more common than *P. pipistrellus*. *Nyctalus noctula *were present on some transects, with only *n* = 18 passes; consequently, *N. noctula* passes were excluded from analysis.

Significantly fewer *M. daubentonii* were recorded on the outgoing leg of each transect (paired *t* test, *n* = 16, *p* < 0.05), comprising 206 contacts on the outgoing leg compared with 350 on the return leg. There was no significant difference (*p* > 0.05) in numbers of both *P. pipistrellus* and *P. pygmaeus* foraging on the outgoing and return legs (96 outgoing, 125 return and 417 outgoing, 391 return, respectively).

**Figure 3:**
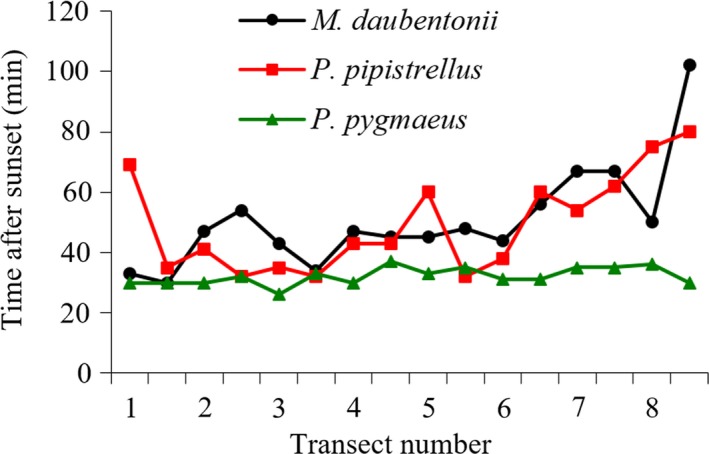
Time of first bat recorded after sunset at each transect, one (low altitude) to eight (high altitude). Transects include replicates *n* = 2 nights per transect (*n* = 16 nights)

Time at which bats were first recorded at each transect varied with species and altitude. *M. daubentonii* was the latest bat to be recorded with a mean ± *SD* (first recorded) of 50.8 ± 16.63 min after sunset. Time for *P. pipistrellus *may be inaccurate because of the smaller sample size (49.9 ± 16.21 min), and *P. pygmaeus* was the first bat to arrive at the foraging sites (mean 32.1 ± 2.82 min after sunset). Time first recorded after sunset increased with altitude for both *M. daubentonii *and *P. pipistrellus* but remained constant for *P. pygmaeus *(Figure 3). The midriver section (transects 3–6) contained the most constant times recorded after sunset for *M. daubentonii* with bats being recorded the earliest at transects three and five.

### 3.3 Habitat selection

**Figure 4:**
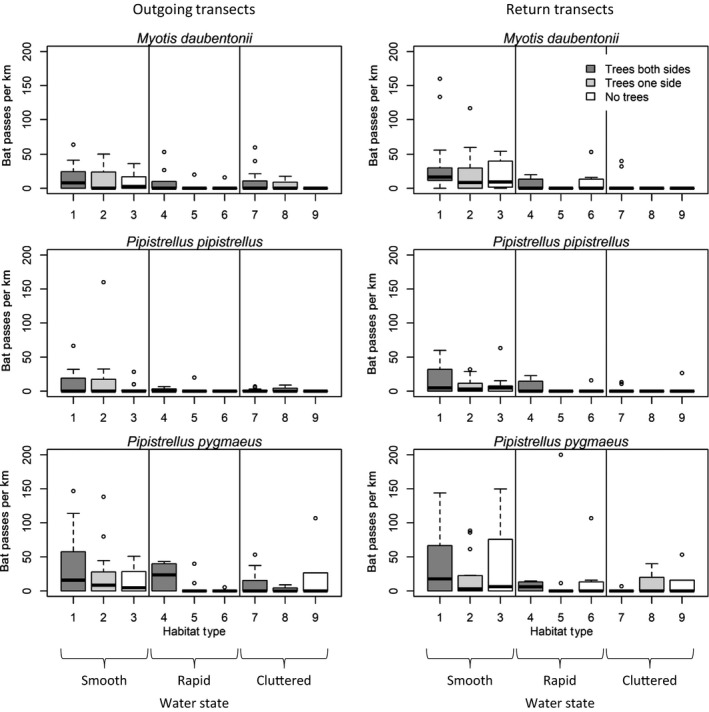
Box plot of number of passes per km of three bat species on outgoing and return transect legs. Box represents interquartile range, and horizontal line is median. The two vertical solid lines simply delineate water surface category

More detections of each species were recorded over smooth water in both outgoing and return transects, particularly in areas with trees on one or both sides of the river (Figure 4). *Pipistrellus pygmaeus *was detected in the highest density (passes km^−1^) in smooth water habitats, but of the three species, it was also most commonly detected in rapid and cluttered areas.

A chi‐square analysis was performed on bat passes in each habitat type. For outgoing and return transects, *M. daubentonii* were not distributed randomly between habitats (outgoing *χ* ^2^ = 49.63, *df* = 8, *p* < 0.0001 and return *χ* ^2^ = 63.40, *df* = 8, *p* < 0.0001). Nor were distributions of *P. pipistrellus* (outgoing *χ* ^2^ = 40.36, *df* = 8, *p* < 0.0001 and return *χ* ^2^ = 42.93, *df* = 8, *p* < 0.0001) and *P. pygmaeus* (outgoing *χ* ^2^ = 101.16, *df* = 8, *p* < 0.0001 and return *χ* ^2^ = 108.03, *df* = 8, *p* < 0.0001). Bonferroni confidence intervals were calculated at the *p < *0.05 level (Neu et al., 1974). These determined whether each habitat was selected more, less, or in proportion to availability than would be expected from a random distribution.

*Myotis daubentonii* showed a significant (*p* < 0.05) preference for stretches of river with a smooth water surface with trees on one bank (Table 4). Smooth water with trees on both sides and with no trees was used in proportion to availability. The latter meant that either bats were not selecting for or against a habitat or that data were insufficient to differentiate between for or against. Overall, rapid and cluttered water categories were either selected against or used in proportion to availability, potentially because bats were obliged to traverse these locations when traveling to targeted (more suitable) foraging locations (Arlettaz, 1999).

**Table 4:** Habitat usage for each species for outgoing and return legs of transects

	Water character					
	Smooth		Rapid		Cluttered	
	Out.	Ret.	Out.	Ret.	Out.	Ret.
Trees						
*Myotis daubentonii*						
Both sides	AA 38 (33)	AA 66 (56)	AA 24 (24)	AA 33 (40)	SA 8 (22)	SA 21 (38)
One side	SF 86 (50)	SF 136 (85)	SA 1 (6)	SA 0 (9)	AA 2 (3)	SA 0 (6)
None	AA 44 (56)	AA 80 (95)	SA 3 (10)	AA 14 (16)	SA 0 (3)	SA 0 (5)
*Pipistrellus pipistrellus*						
Both sides	AA 25 (15)	SF 41 (20)	SA 4 (11)	AA 9 (14)	SA 4 (10)	SA 3 (14)
One side	SF 43 (23)	AA 34 (31)	AA 1 (3)	SA 0 (3)	AA 1 (2)	SA 0 (2)
None	AA 18 (26)	AA 36 (34)	SA 0 (4)	SA 1 (6)	SA 0 (1)	AA 1 (2)
*Pipistrellus pygmaeus*						
Both sides	SF 121 (66)	AA 52 (62)	AA 54 (48)	SA 12 (45)	SA 23 (45)	SA 2 (42)
One side	SF 128 (102)	SF 137 (95)	SA 3 (11)	AA 11 (11)	SA 1 (7)	SA 1 (6)
None	SA 81 (113)	SF 151 (106)	SA 1 (19)	AA 22 (18)	AA 5 (6)	AA 3 (6)

Note

Out. = outgoing transect, Ret. = return transect. Assuming bats are detected evenly in all habitats, cells that were Selected For (SF = green) represent habitats with a higher than expected number of bats detected within them and Selected Against (SA = red) represent habitats that were selected against with a Bonferroni *p*‐value of <0.05. White cells represent those that were occupied proportional to their availability (AA). The values in each cell show number of bats detected in each habitat and in parentheses, number expected.

*Pipistrellus pipistrellus* showed a mixture of preferences, generally selecting for smooth water with either trees on one or both sides and avoiding or showing no preference toward rapid and cluttered water stretches (Table 4). *Pipistrellus pygmaeus *again showed a mixture of preferences for habitat mainly selecting smooth water with trees (and in one case without trees) and avoiding rapid and cluttered water (Table 4).

**Figure 5:**
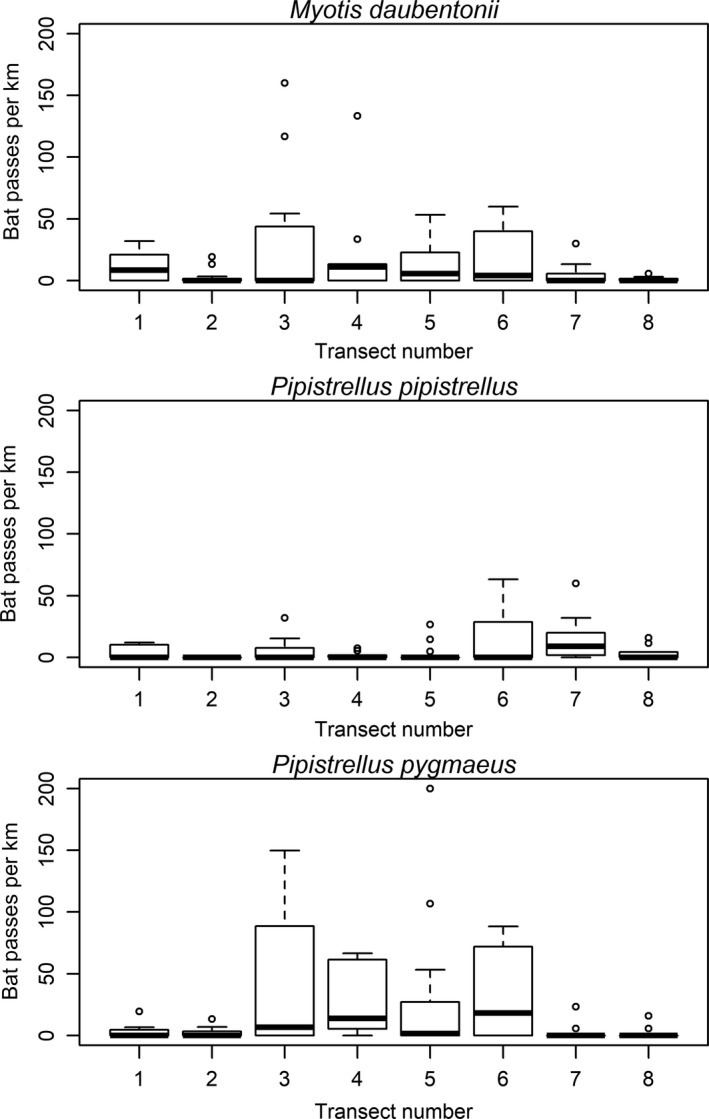
Box plots of bat passes per km of the three bat species at each transect. Box represents interquartile range, and horizontal line is median

A chi‐square analysis was performed on counts of bats in each transect for return transect data only. None of the three species were distributed randomly between transects (*M. daubentonii* *χ* ^2^ = 149.91, *df* = 7, *p* < 0.0001; *P. pipistrellus* *χ* ^2^ = 326.49, *df* = 7, *p* < 0.0001 and *P. pygmaeus* *χ* ^2^ = 283.99, *df* = 7, *p* < 0.0001). Detections of both *M. daubentonii* and *P. pygmaeus* were highest along the middle section of the river (Figure 5; transects 3–6). These transects were at an elevation of 130–180 m and contained the most habitat two (smooth water with trees on one side) along the Lledr. There were comparatively few recordings of *P. pipistrellus *in the lower and middle sections of the river; instead, they were detected most commonly in transects 6 and 7, which are at higher elevation (180–207 m) and feature primarily smooth water habitats.

## 4 DISCUSSION

### 4.1 Bat detections

All three species of bat (*M. daubentonii*, *P. pipistrellus* and *P. pygmaeus*) were detected over the Lledr at some point in the early evening and the proportion of time spent in different habitat types varied between species.

Detection of *M. daubentonii* was reasonably high (*n* = 556 counts). Siemers, Stilz, and Schnitzler (2001) propose that *M. daubentonii *forage low over the surface of smooth water, as it enhances acoustic detection of prey. Todd and Waters (2017) also report that *M. daubentonii* from the same region as this study are detected most often over smooth water in areas with trees on one or both sides, and slightly less over smooth water with no trees.

The lower activity of *M. daubentonii* on the outgoing leg of transects in comparison with the return may be explained in terms of bat emergence, roost location, and habitat. *M. daubentonii* arrived on average 20 min later than *P. pygmaeus*. Mean emergence times after sunset for *M. daubentonii* from previous studies are 73 min (Jones & Rydell, 1994) and 40 min after sunset (Warren et al., 2000).

*Pipistrellus pipistrellus *was the least common species in this area (*n* = 221 passes). This species may be less abundant in this area or may not be as dependent on riparian habitat. Other studies have reported this species using other types of habitat to forage after emergence (Racey & Swift, 1985; Rydell, Bushby, Cosgrove, & Racey, 1994; Todd & Waters, 2017).

*Pipistrellus pygmaeus *was the most frequently detected species (*n* = 808 counts) indicating that riparian habitat in this area may be important for this species. This result complies with findings of Barlow (1997), who suggests that this species is associated with water and insects which have aquatic larval stages. Habitat preferences for *P. pygmaeus *are considered to be different than those of *P. pipistrellus*, with the former detected more frequently in riparian habitats and the latter detected in a wider variety of habitats, as also reported by Walsh and Harris (1996). This study has revealed that the Lledr valley is a potentially suitable foraging area for *P. pygmaeus*, which in Wales, may be at risk from climate change, since higher winter temperatures between January and March have been linked to previously a smaller population size (Andrews, Crump, Harries, & Andrews, 2016).

The roughly equal activity of *P. pipistrellus* and *P. pygmaeus* on both outgoing and return transects is likely related to emergence time from roosts. For pipistrelles, mean emergence times after sunset have been reported to be between 21 min (Jones & Rydell, 1994) and 33.5 min (Bullock, Combes, Eales, & Pritchard, 1987). Transects in this study were begun 30 min after sunset, which suggests that the lack of difference in detections on outgoing and return transects is because the bats have already emerged and may already be at their foraging sites at the beginning of the transect. This is common for pipistrelles, which are often reported to roost close to major rivers (e.g., Speakman et al., 1991). Arrival times of *P. pygmaeus* at the river showed little variation (range 11 min), suggesting that this species might head straight for the river upon emergence. *Pipistrellus pipistrellus* arrival times were variable (range 48 min) suggesting that they either emerge later than *P. pygmaeus* or *P. pipistrellus* may head to other areas after emergence and not necessarily the river.

Previous studies by Walsh et al. (1995) and Walsh and Harris (1996) have measured mean number of bat passes per km in marginal upland areas to be 5.5 and 6, respectively. This study, which used the same methodology as Walsh and Harris (1996), recorded a much higher number of passes per km (mean ± *SD* of 39.6 ± 62.67) of all species using the return transect data (pooled for all transects). Figures as high as 160 bat passes per km for *M. daubentonii* (14.5 ± 28.96 bats/km) and 160 (5.8 ± 11.95) and 227 (18.5 ± 37.13) passes per km for *P. pipistrellus* and *P. pygmaeus*, respectively, were recorded in the area. All highest counts were recorded within the categories of smooth water with trees on both or one side, which further places importance on these habitat types within a marginal land class. These counts are comparable to those given by Warren et al. (2000) of a total mean of 20.1 passes per km for all bats, 8.9 passes per km for *M. daubentonii,* and 11.2 passes per km for *P. pipistrellus*, although their five passes of *P. pygmaeus* are too low to present as a mean value. Indeed, numbers of bat passes per km of all land classes in the Walsh et al. (1995) and Walsh and Harris (1996) studies are far lower than those recorded here and by Warren et al. (2000) in Wharfedale, Yorkshire, UK. In comparison with Wharfedale, the Lledr valley is more heavily wooded. There may potentially be more roosts available for bats, and therefore, the area may support a larger population of individuals than Wharfedale (if roosts, rather than food are a limiting factor), though no population estimates can be made from bat passes alone.

### 4.2 Habitat selection

Distribution of all three species reflected a mixture of habitat availability, type, and elevation. Along the river Lledr, the only habitat types that bats selected for were the smooth water habitats and for each species, smooth water with trees on one bank accommodated a higher number of bat passes than would be expected in a random distribution (Table 4). *Pipistrellus pygmaeus* was the only species to select for smooth water with no trees on the return transect; however, this same habitat was selected against on the outgoing transect. Rapids and cluttered water categories were either used as available or selected against by each species. These findings are similar to the findings of Warren et al. (2000), Todd and Waters (2007), and Todd and Waters (2017).

In general, there were fewer bats present at both extremes of the river (low and high), with a distinct clumping of bat detections in the midelevation range. The lower Lledr sections, in contrast to middle and higher elevations, are steeper with more fast‐flowing water, while the relatively flat areas in the midriver section are more reliable in terms of their surface water state in differing weather conditions (V. L. G. Todd, personal observation). *Pipistrellus pygmaeus* appears to take advantage of the most diverse habitats in this area (Figure 4).

Suitable habitat (smooth water with trees on one or both sides) was readily available in transect seven, yet there were far fewer detections of *M. daubentonii* and *P. pygmaeus* than in transect 6 which was nearly 30 m lower in elevation. It may be that although sufficient habitat is available at higher elevation, other factors such as lower temperature and less predictable insect availability restrict bats in these areas (Capaverde et al., 2018; Dunn & Waters, 2012; Nardone et al., 2015; Todd & Waters, 2007). Indeed, foraging activity of bats and insect availability has been shown to decrease with increasing elevation (Grindal & Brigham, 1999), as has bat species richness (Patterson, Stotz, Solari, Fitzpatrick, & Pacheco, 1998); however, detections of *P. pipistrellus *were higher in transects six and seven than in lower transects.

de Jong and Ahlén (1991) found the distribution of pipistrelles to be correlated with local insect abundance, but there are conflicting reports of numbers of insects in relation to water smoothness. Warren et al. (2000) reported that numbers of insects increase with the smoothness of the water, whereas Todd and Waters (2017) found that insect distributions were independent of water surface state. Rydell, Miller, and Jensen (1999) reported that aerial insects were more abundant over ripples, so insect distribution could not explain why bats in this study avoided those habitats. Insect abundance has also been found to be unrelated to the presence of bank‐side vegetation (Todd & Waters, 2007), which might explain why *P. pygmaeus* also selected for the smooth water category with no trees. Selection of trees during the twilight period may permit earlier emergence to exploit the dusk insect peak. Bats may use trees as part of a predator avoidance behavior when they may be particularly susceptible to predation by crepuscular birds (Mikula, Morelli, Lučan, Jones, & Tryjanowski, 2016; Speakman, 1991). Observations suggest that bats are most frequently attacked by raptors just after evening emergence and as they travel to and from the roost (Fenton et al., 1994; Mikula et al., 2016). This would explain pure selection of trees on outgoing transects (30 min after sunset) when it is lighter and selection of areas with no trees once the threat of predation has diminished.

Transect eight (the highest elevation) had plenty of habitat three (smooth water, no trees), but there were very few *P. pygmaeus*. Again, effects of physical variables associated with elevation (high wind, temperatures that are more variable, and less vegetation cover) might be overriding bats’ ability to reliably utilize suitable habitat types.

## 5 CONCLUSION

A changing climate has been reported to cause alterations to bat detection, habitat use, reproduction, and migrations (Adams, 2010,2018; Rebelo et al., 2010; Sherwin et al., 2013); therefore, while data presented in this study were collected 20 years ago, they provide a useful snapshot of the detection of bats in the Lledr valley during the summer period. This can be used as a baseline to which future data can be compared to investigate potential effects of a changing climate and or land development on these species. All species were recorded more in areas with moderate elevation and smooth water; however, there was suitable smooth water habitat available at higher elevations which was unoccupied. It is possible that bats could occupy these higher sites in future, especially if anthropogenic pressures, such as land development in other sections of the river, marginalize bats into using suboptimal habitats.

In marginal upland river valleys (up to *ca*. 200 m), river management procedures should include (wherever possible) maintenance of smooth stretches of water with trees on either one or both banks. This serves to increase prime habitat areas for *M. daubentonii*, *P. pipistrellus*, and *P. pygmaeus*. Areas above this elevation in the Lledr valley may be less important for foraging bats. These small‐scale altitudinal differences in habitat selection should be factored into design of future bat distribution studies and taken into consideration by conservation planners when reviewing habitat requirements of these species in Welsh river valleys, and elsewhere within the United Kingdom.

## CONFLICT OF INTEREST

None declared.

## AUTHOR CONTRIBUTION

Victoria Todd involved in data collection, interpretation, and manuscript preparation. Laura Williamson involved in manuscript preparation.

## Data Availability

Data have been archived in Dryad. DOI: https://doi.org/10.5061/dryad.2f253b0. Data files: Todd & Williamson 2019 Habitat use of three species of bat.
